# A reflection on ethical and methodological challenges of using separate interviews with adolescent-older carer dyads in rural South Africa

**DOI:** 10.1186/s12910-019-0383-9

**Published:** 2019-07-08

**Authors:** Dumile Gumede, Nothando B. Ngwenya, Stella Namukwaya, Sarah Bernays, Janet Seeley

**Affiliations:** 1 Africa Health Research Institute, Durban, South Africa; 2 School of Applied Human Sciences, University of KwaZulu-Natal, Durban, South Africa; 3 Medical Research Council/ Uganda Virus Research Institute and LSHTM Uganda Research Unit, P.O Box 49, Entebbe, Uganda; 4 Sydney School of Public Health, The University of Sydney, 324, Edward Ford Building A27, Sydney, Australia; 5 Departsment of Global Health and Development, London School of Hygiene and Tropical Medicine, London, WC1E 7HT UK

**Keywords:** Dyads, Adolescents, Older carers, Confidentiality, Ethics, Separate interviews

## Abstract

**Background:**

This article discusses our reflections on ethical and methodological challenges when conducting separate interviews with individuals in dyads in the uMkhanyakude district, South Africa. Our work is embedded in an ethnographic study exploring care relationships between adolescents and their older carers in the context of a large-donor funded HIV programme. We use these reflections to discuss some of the challenges and present possible management strategies that may be adopted in conducting dyadic health research in resource-poor settings.

**Methods:**

Drawing from the relational agency, three rounds of separate interviews and participant observation were undertaken with dyads of adolescents aged between 13 and 19 and their older carers aged 50+ from October 2017 to September 2018. A reflexive journal was kept to record the interviewer's experiences of the whole research process. We identified methodological and ethical challenges from these data during the thematic analysis.

**Results:**

A total of 36 separate interviews were conducted with six pairs of adolescent-older carer dyads (*n* = 12 participants). Five themes emerged: recruitment of dyads, consenting dyads, confidentiality, conducting separate interviews with adolescents and older carers, and interviewer-dyad interaction. We also illustrated how we dealt with these challenges.

**Conclusions:**

Results from this study can guide the recruitment, consenting and collecting data for health studies that employ a similar form of enquiry in LMICs. However, ethical and methodological challenges should be recognised as features of the relationships between cross-generation dyads rather than weaknesses of the method.

## Background

Qualitative studies on dyads have increased [1] since the dyadic research approach emerged in the 1970s in marriage and family studies [2]. The approach examines ‘the dyadic perception of reality, dyadic meaning, and dyadic being-in-the-world, in addition to the relationship component’ [1]. For some research questions, interviewing dyads may generate data that could not be obtained from interviews with individuals [2]. Scholars have used the dyadic approach in studies involving couples [2], caregivers and children or grandchildren [3–6] and patients and carers [7–10] in health research. For example, a Ugandan study examined care dyads of caregivers and HIV-positive young people about their experiences of disclosure of HIV status and the influence it had on their relationship [5]. Different interviewing techniques have been used to collect data with dyads. The interviews can be conducted separately with each dyad member [11] or jointly with both members together [12, 13]. Separate interviews enable each individual to respond from their own perspective, ‘capturing the individual within the dyad, without forgoing the dyadic perspective’ [1], whereas conducting interviews jointly results in a shared narrative [14]. A separate interviewing approach raises methodological challenges related to the method of conducting interviews with each individual member of a dyad and its influence on the data collected [14]. Separate interviews can be time-consuming for the dyad members and the researcher because two interviews are carried out [15]. It also raises ethical concerns related to the personal relationship between dyad members when they are interviewed separately [16]. Therefore, conducting separate interviews with dyad members could pose potential methodological and ethical problems that should not be overlooked.

Methodology and ethics are interrelated. Adherence to ethical standards can add to the value of research and, conversely, methodological soundness can strengthen ethics [17]. In their editorial article, Knottnerus and colleagues [18] explain the link between methodology and ethics that ‘ethics of research methodology requires a methodology of research ethics’. Reviewing the ethics of research should consider whether the research questions are worth asking and if the methods used are an effective way of answering them [17]. The approach of conducting separate interviews as a methodology has ethical implications as it critically influences data collection, interpretations of data and reporting of data.

Ethical issues arise in all aspects of research and are particularly salient when studying vulnerable populations such as children and adolescents, older people and people living with HIV or affected by HIV [19–21]. Researchers have ethical responsibility for ensuring that individuals are given all the information needed to make informed decisions about whether to participate in research or not. The process of respecting autonomy is complex, given different conceptions of personhood [22]. Most persons define themselves and make decisions within a wider network of social relationships [22, 23]. When engaging with an individual, one is, in fact, dealing with a complex relational web of persons who may include the immediate family, peers and significant others for whom the decision matters [22]. However, to respect autonomy, Osamor and Grady [23] suggest researchers need to understand and respect relationships that are important to individuals and the process with which they incorporate the values inherent in these relationships into their decision-making. Adolescents may agree to participate out of obedience to or respect for their caregivers [19, 20]. For example, in a study to understand the resolution of discordance between adolescent-parent dyads about participation in research, Francis and colleagues [24] report that sometimes one individual in the dyad asserted authority over his/her partner about the decision for participation.

Confidentiality is one of the cornerstones of research involving human participants. Protecting research participants’ right to confidentiality is a responsibility shared by researchers, institutional review boards, and participants themselves. However, combining ethnographic and dyadic approaches adds further complications to maintaining the confidentiality of individuals within the same dyad. It is difficult to maintain confidentiality between the members of the dyad when information is validated, or when different versions are compared [1]. The challenge of confidentiality also arises when reporting findings on the dyad as the amount of information that could identify dyad members could be more than in individual interviews [16]. Separate interviewing has the potential to generate anxiety within dyads because this approach might suggest that secrets exist, and that one person is willing to share these secrets with the researcher (and not with his or her partner in the dyad) [14]. Individuals may express the desire to know what the other person in the dyad said, placing the researcher in an awkward position [25, 26]. The method of separate interviewing can confront the researcher with a dilemma of how to make sense of different versions of a story regarding events in individuals’ lives [11]. Furthermore, in the face of competing accounts from individual interviews, the researchers are limited in their ability to probe further and ask direct questions, since in so doing, they may unintentionally disclose what the other individual said, thereby breaching confidentiality [11, 25, 27].

There may be an expectation of short and long-term benefits and advantages to participation that influences individual consent and participation. This challenge may be greater when doing ethnographic work among vulnerable populations, because of the extended amount of time spent with participants [20]. In a project with children and women affected by HIV/AIDS in Kenya, Nyambedha [20] found that the people in the area did not differentiate between activities of non-government organizations (NGOs), other researchers and his own study. Consequently, there were raised expectations that participation in the research could lead to interventions that would assist participants. Nyambedha argues that researchers can cause harm if no action is taken to address the high expectations participants may have.

As shown, researchers face complex questions of methodology and ethics in the application of a separate interviewing approach. These challenges are of a complex nature, yet dyad studies have received a relatively small amount of critical ethical and methodological attention [12, 16, 27, 28], particularly in low-middle-income countries (LMICs).

In this article, we draw upon our experience of conducting separate interviews in an ethnographic project with care dyads of adolescents and their older carers in rural South Africa and examine the ethical and methodological challenges, which have arisen from our work and how these were addressed. We use the reflections to discuss some of the challenges and present possible management strategies that may be adopted in conducting dyadic health research in LMICs.

### Research design, method and sample

The study employed an ethnographic approach to explore care relationships between adolescents and their older carers in South Africa, and the contextual factors associated with HIV risk among these adolescents. Historically, the care of orphaned and vulnerable children and adolescents in Africa is often provided by older carers in extended families [29]. The onset of the HIV epidemic left many adolescents orphaned, vulnerable and in need of care [29, 30]. In many cases, those who fill the care deficit are older women [30]. The increased caring responsibilities by older carers commonly coincides with a time when many older people themselves need care. Older carers may experience health problems due to age [31, 32] and caregiver stress [29], which impacts negatively on the care relationship and the kind of care given [31]. It is important to understand how adolescent care is provided by older carers and experienced to improve the kind of care that adolescents receive, and indeed, the reciprocal care the older person may benefit from.

The theoretical framework of relational agency informed the research question, methods and interpretation of findings. Relational agency demonstrates that individuals’ experiences are shaped and influenced by their relationships with others [33]. Relational agency refers to how the ability of someone to exercise agency is bounded by their relationships, as well as the structures of their environment. Their relationships, which are dynamic and evolving, are crucial structures within their environment. In adolescence, it may be a particularly useful theoretical construct because of the fluidity and rapidly changing nature of young people’s relationships. The influential relationships, which are guiding their behaviour, are changing too as social and peer relationships and influences proliferate.

The increasing attention paid to relational agency in shaping adolescent’ experiences and health-seeking behaviour shows there is clearly a need for more dyadic research, which will provide insights into these relational experiences, challenges and solutions that should directly benefit the provision of effective support for both adolescents and their older carers in LMICs. In addition to capturing the young people’s perspective, understanding how the relationship between the older carer and young person is experienced and how this affects care and well-being is important. The dyadic approach to interviewing offers considerable potential to explore how relationships of individuals in a dyad influence data collection and ethics processes.

The study was conducted at the Africa Health Research Institute (AHRI), in the uMkhanyakude district of northern KwaZulu-Natal, South Africa, in the context of a large donor-funded programme in KwaZulu-Natal, South Africa. Different implementing organisations delivered the programme activities in the uMkhanyakude district to address HIV risk behaviours, HIV transmission, and gender-based violence.

We purposively sampled adolescent-older carer dyads to achieve an in-depth understanding of the participants’ experiences. Participants were selected according to the following criteria:

- An adolescent girl or boy aged between 13 and 19 years living and cared for by an older carer (either older man or older woman) aged 50+ years in the uMkhanyakude district.
- An adolescent who was the recipient of at least one HIV behavioural intervention from the selected implementing organisation.
- An older carer who was the primary caregiver for an adolescent girl or boy who was eligible based on the above criteria.

An implementing organisation provided permission to conduct the study and to recruit its recipients through programme facilitators. The programme facilitators applied the inclusion criteria to identify dyads to be invited to participate in the study.

The first author, a woman with experience in qualitative interviewing, conducted participant observation and three separate interviews with six adolescents (aged 13–19) and six older carers (aged 50+) from October 2017 to September 2018. In total, thirty-six interviews were conducted. We adopted a separate interviewing approach with each participant being interviewed one on one. Conducting the interview in private was a priority. Even if the other individual in the dyad was also at home, the interview was conducted in a separate space without anyone else being able to overhear. The interviewer used a similar interview guide with each person to explore issues about the participants’ background, relationship with older carer/adolescent, parenting practices, communication about sexual and reproductive health, and experience with interventions. However, we adjusted questions to be more appropriate for different ages. The interviewer also visited dyads at home to conduct participant observation at different times of the day. These data were recorded in field notes focusing on what was observed, informal conversations with participants, records of activities, conversations between participants, and participants’ nonverbal and verbal behaviours.

The first author kept a reflexive journal in which she noted her experiences of the whole research process. The journal contained concrete descriptions of the interviewer’s experiences about ethical and methodological challenges emerging in this dyad study of care relationships between adolescents and their older carers and how these were addressed.

Interviews were audio-recorded, transcribed and translated into English. Transcripts and field notes were coded and managed using NVivo 11. Following dyadic analysis, we examined themes emerging from each dyad member’s narratives [1] by assessing contrasts and overlaps between the individual versions. This paper does not aim to report on the substantive findings of this analysis, but rather we focus on ethical dilemmas emerging in this dyadic study of care relationships between adolescents and their older carers. We identified methodological and ethical challenges from these data during the thematic analysis.

### Ethical considerations

The study received approval from the University of KwaZulu-Natal Humanities and Social Sciences Research Ethics Committee (ref HSS/1109/017D). We obtained written informed consent from all participants before they took part in the study. For adolescents aged less than 18 years, we obtained written consent from their older carers and written assent from the young person themselves. No names of participants were recorded; instead, participant codes were used, and the names of organisations anonymised.

## Results

### Participant profile

Six adolescent-older carer dyads (*n* = 12 participants) took part in this study. All the six older carers were women aged between 56 and 80 years and caring for between two to 15 grandchildren. Two were paternal grandmothers, and four were maternal grandmothers. This reflects the gendered nature of caregiving common in this setting. Some older carers started caring for grandchildren since childbirth, while others assumed caregiving responsibilities when grandchildren in-migrated to the older carer’s households. The number of years as carers ranged between three and 14 years. All older carers relied on either a state pension or child support grant for a source of income. Of the six older carers, only one was cohabiting with a sexual partner; while others were widowed. Only two older carers had attended primary school. Although not questioned directly about their HIV status, two older carers disclosed that they were living with HIV and on ART.

There were five adolescent girls and one adolescent boy in the study, between the ages of 13 and 19 years. Four were still in secondary school while the other two had dropped out of school due to pregnancy and academic difficulties. Of the six adolescents, all were recipients of either one or two HIV prevention programmes from a single implementing organisation. All adolescents’ biological mothers were still alive, and four were paternal orphans. There were various reasons why the biological mothers were not providing primary care to the adolescents including maternal abandonment, non-marital births, unemployment and migration.

### Ethical and methodological challenges

The information recorded in the first author’s journal highlighted several ethical and methodological challenges, which guided the choice of the following themes: recruitment of dyads, consenting dyads, confidentiality, conducting separate interviews with adolescents and older carers, and interviewer-dyad interaction, and how these challenges were addressed in the study. It must be noted that some challenges were a combination of both the method and ethics. We have thus indicated whether each challenge is a methodological, ethical or mixed challenge.

### Recruitment of dyads

#### Potential sampling bias

We anticipated that recruiting participants through the implementing organisation had potential sampling bias prior to the recruitment process. The programme facilitators relied on their self-knowledge to identify potential participants, as the facilitators were local community members themselves. Although this was an effective way of identifying participants, there was potential for bias about individuals that the programme facilitators chose to approach. Given that the study involved exploring participants’ experiences with HIV behavioural interventions, programme facilitators might have been tempted to lean towards inviting people who were thought likely to provide a more positive account of the organisation and the interventions than others. To reduce potential sampling biases, we worked with two different programme facilitators for each to identify two pairs of adolescents and their older carers. We also integrated a participant-driven sampling technique by requesting two adolescent participants to refer the researcher to other adolescent recipients in the care of older people. Having a small sample of participants who know each other in the neighbourhood raised further concerns about maintaining the confidentiality of results. To keep the dyad partners’ versions confidential from each other, we only checked our interpretations with individuals, treating them as separate sources. To enhance the anonymity and confidentiality of individuals, we needed to omit or slightly alter some distinguishing information, which could identify individuals from another [1]. We did data omissions and alterations to balance the need to preserve both contextual issues and confidentiality [1]. For example, we either omitted or altered information such as names of birthplaces, names of schools and church names. In addition, we provided all participants with a pseudonym to ensure confidentiality and to protect the privacy of the individuals.

#### Risk of coercion to participate

An ethical challenge that emerged from the recruitment of dyads was the risk of coercion to participate. Firstly, we suspected that there was potential coercion during the recruitment as some individuals may have felt under some obligation to participate if approached by programme facilitators. It was even more concerning when the researchers learnt that the facilitators were local community members, having established relationships with potential study participants in the area. For example, one of the facilitators was a pastor’s wife and running a local crèche. It was highly probable that some individuals were members of the same church with the facilitator. We were concerned that some individuals were likely to feel obliged to participate in view of maintaining the existing relationship with the facilitator by not refusing to participate. Secondly, older carers knew their adolescent grandchildren participated in HIV behavioural interventions. Approaching older carers first and for them to provide initial agreement may have had implications for how ‘free’ adolescents were to then refuse to participate. Similarly, approaching adolescents first could have been perceived as disrespectful to the older carers. We were concerned about the potential of members influencing or coercing each other for participation in the study. An option for approaching them together was difficult as it was not easy to find them both at home at the same time. Older carers were more accessible to contact and meet, given that they were mostly at home, as they were unemployed and spent their time doing household activities. Whereas, adolescents were more difficult to contact as they were frequently away from home. All potential approaches involved compromises and risks. However, the implementing organisation guided us as to the most appropriate strategy to reach the participants. In all cases, the older person was contacted first and then efforts made, on approaching the young person, to make them at ease and able to make their own decision about whether to take part. The programme facilitator and the interviewer visited the individuals together at home for an introductory meeting; thereafter the interviewer was left with the individuals to discuss the study and to seek consent for the older carer and their adolescent grandchild. The interviewer emphasised voluntary participation to the adolescents in view of reducing the risk of coercion by the older carers and the programme facilitators. All the six dyads approached, consented to participate in the study. There were no refusals to participate or withdrawals from the study.

#### Non-contactable dyad individuals

Recruiting dyad individuals was time-consuming given that adolescents were often away from home, so repeat visits were required. In addition to school-related activities, young people were involved in doing household chores outside of the home such as fetching wood and water, attending church-activities or visiting family relatives. Both members had to be first contacted and invited for participation prior to consenting and interviewing. Failure to contact the other member halted the process until both consented as we did not know if the other member would agree to participate or not. Commonly, the older carer assisted the researcher with information about when the adolescent was likely to be home and contactable. This required repeat visits until contact was made with an adolescent. Nonetheless, the interviewer established a stronger rapport with older carers during those repeat visits, which somehow influenced unintended preferential treatment given to some participants by the interviewer. This will be explained further in the section discussing the challenges of interviewer-dyad interaction.

### Consenting dyads

#### Differing needs of dyad members

The process of consenting dyads was both ethically and methodologically complex. Differing needs of individuals in dyads challenged the provision for informed consent and assent during the process. Some participants considered the study information sheet to be too long. All the older carers wanted the sheet read to them because they could not read themselves due to literacy skills or vision problems. Some non-literate older carers were not comfortable signing the consent form. In contrast, the adolescents did not want to spend the time reading the information sheet through and preferred the researcher explain it to them. They wanted the consent process to be accelerated to fit in with their limited time that they were willing to devote to the research given their competing commitments. As a result, we adapted the informed consent and assent process to the needs of individual dyad members such as reading or paraphrasing the sheet and the use of a mark in place of a signature on the consent document. However, rushing the process of informed consent raised the dilemma of being uncertain about participants’ full understanding of informed consent. We ensured that the interviewer presented the study information to the study participants at every repeat interview and confirmed with the participants if they were still willing to continue participation in the study.

#### Parenting style

We observed different parenting styles among older carers during the process of informed consent. Some older carers demanded total control of the process and expected children to obey their decision about research participation, while others encouraged adolescent autonomy in research participation. For example, B1 (female older carer aged 64) consented to participate in the study and the interviewer informed her that B2 (adolescent girl aged 14) was also required to decide on her own about participation in the study. In her response, B1 told the interviewer not to bother because she would instruct B2 to participate. Knowing that valid informed consent was essential to enable individuals to be fully aware of what they were taking part in, the interviewer took time to explain the principle of voluntary participation until the older carer understood and linked this to their knowledge of the political changes in South Africa about children’s rights. In contrast, older carer E1 (female older carer aged 56) expressed that she was happy for E2 (adolescent girl aged 13) to make her own decision to participate or not because, although regarded a child, she was capable of doing so.

### Confidentiality

#### Participants’ limited trust in the confidentiality process

Some participants displayed limited trust in the confidentiality process, concerned that information might get to the other dyad member or even if it did not, there were concerns about being seen to voice criticism about the other member of the dyad. To illustrate this, we draw two examples from the field notes. In the first example, an adolescent girl was uncertain about disclosing confidential information to a stranger, hence the adolescent wanted assurance that the interviewer would not share information with her grandmother:

“Today, I had the first interview with C2 (adolescent girl aged 15) who lived in the care of her 80-year old grandmother (C1). Prior to this interview, I had already interviewed C1 on the previous day, where she narrated her perspective of her relationship with C2. Amongst her concerns, C1 suspected that C2 was pregnant. Therefore, I went to interview C2 having this confidential information that her grandmother and others in the family were suspecting that C2 was pregnant. During the interview, while I was keen to find out from C2 about the suspicions of her pregnancy but I did not ask her about it. She also did not talk about it until towards the end of the interview, when I asked her if she had any other thing she wanted to tell me before we closed the interview. She started mumbling, facing down and hesitant to talk. I motivated her to feel free to talk to me. She looked straight into my eyes, and asked, ‘*are you going to tell my grandmother that I am pregnant?*’ This was her secret, as she was not planning to tell her grandmother. She preferred her grandmother to discover the pregnancy on her own; otherwise, she was nervous she could be expelled at home if her grandmother discovered the pregnancy in its first trimester. As a result, she wanted assurance and commitment from me that her secret would not be passed on to her grandmother”.

In the second example, an older carer was hesitant to narrate confidential information about her grandson and his mother because she feared that her grandson might learn if the interviewer did not keep the information confidential:

“My second interview with A1 (older carer woman aged 64) revealed that A2 (adolescent boy aged 15) was not happy about using his maternal surname hence he was in a process of changing to his paternal surname. When trying to understand the reasons for changing the surname, before she could answer A2 raised up her hands (as if surrendering) and said: ‘*it’s difficult to explain people’s secrets [grandson and his mother] … please protect me by keeping this as a secret*’. She wanted a guarantee that her grandson would not find out that she divulged his secrets to me”.

Both A1 and C2 had limited trust in the confidentiality process and needed assurance. They were concerned that breach of confidentiality would result in a conflict within the relationship.

To enhance individuals’ trust and confidence in the confidentiality process, we employed several strategies. Firstly, at every start and end of interviews, the interviewer consistently assured each individual that the researcher would not disclose information to the other dyad member. Secondly, we also secured individuals’ confidence through the interviewer’s conduct of adhering to the principle of respect for individual confidentiality by not disclosing information between dyads. Lastly, the nature of repeat interviews allowed the trust to develop which had been earned through their experience of the first interview.

#### Fear of unintended disclosure of information by the interviewer

There was no accidental disclosure of information by the interviewer to dyad members; however, there was fear for the interviewer to mix up individual stories between adolescents and their older carers. Mixing up information could have led to unintended disclosure of information. To avoid any unintended disclosure, before conducting subsequent interviews in the same dyad, the interviewer carefully read and reread the summary and listened to the audio of the member’s previous interview to recollect interview content with that member. The interviewer also took rigorous reflexive journal notes throughout the data collection process to maximise awareness of potential threats to confidentiality and continuously reflected on best practice to protect each member’s confidentiality. This enhanced adherence to confidentiality by guarding against slippages.

#### Right to access confidential information of the other dyad member

It was not only about the researcher not disclosing information unintentionally but also managing requests for breaching confidentiality by a participant. Some participants, particularly older carers, expected the interviewer to inform them about issues discussed with adolescents. For example, after interviewing C2 (adolescent girl aged 15) at home, C1 (older carer aged 80) immediately approached the interviewer to demand information about her adolescent granddaughter from the interviewer. She was suspicious and wanted to find out from the interviewer if the adolescent was indeed pregnant. The older carer wanted to overrule confidentiality and be given the right to access information because of her expectation of authority over the adolescent. This complicated the dyadic research because the interviewer was trying to retain their respect and trust while disagreeing with and not complying with the carer’s request for information. The interviewer politely reminded participants about the confidentiality clause and affirmed that by so doing she wanted all participants to trust her about their information. This approach appeared effective because attempts to seek information about the other member stopped.

### Conducting separate interviews with adolescents and older carers

#### Expectations of benefits for research participation

Spending extended periods of time with the same group of participants generated expectations of benefits among the participants for their participation in the study. As the participants were narrating their problems to the interviewer, they hoped for solutions from the interviewer. It was the older carers who had raised expectations of assistance, although one adolescent girl (C2) who was requesting for a loan on behalf of her grandmother (C1) to settle a financial debt as their lives were under threat. The older carers expected assistance with employment opportunities, food, housing, medication for arthritis and minor ailments, counselling, money, and adult schooling opportunities. It was interesting to note that the expectations of benefits were required to address their relational needs. For example, older carers repeatedly reported the challenges of caring for their adolescent grandchildren and their concerns about the adolescents’ risky behaviours. Participants mentioned poor communication between the older carers and the adolescents as a challenge in the relationship. The older carers often requested the interviewer to intervene in strained dyad relationships and expected the interviewer to provide some form of counselling. Being caught in the middle of strained relationships between dyad members was stressful for the interviewer, as she did not anticipate the magnitude of relationship problems yet could not help.

Several measures were taken to address these concerns. First, all the participants were offered snack packs as a token of appreciation at every visit. The participants appreciated the packs, as they would share the items in the family. Second, the interviewer continued to explain her role as a researcher, not a therapist, in accordance with the study protocol. However, to compensate for the researcher’s inability to help, participants were provided with a self-referral list with contact details of local service providers, which they could contact for support, and the responsibility to contact the service providers was left with the study participants. This raised an ethical concern that the information support that we provided for self-referrals was very difficult for our participants to then take forward in this setting. For many study participants, a lack of financial resources was a barrier for them to access services due to related costs such as transport. Mostly older carers were non-literate and could not make phone calls, and others did not have access to telephones to contact service providers. Participants could not understand why the interviewer did not contact service providers on their behalf, as they did not have resources to do so. We were concerned that participants were likely to lose interest in the study if they thought researchers were not supportive. Ideally, facilitating the linkage between study participants and the service providers would have been the best solution; but this had budgetary implications, which were not covered in the study. Our strategy was to spend more time explaining the role of research, thereby emphasising its remit. This approach appears to have ameliorated misunderstandings, but it illustrates the challenges of conducting such relationally focused research in low-income settings.

#### Contradictory stories

Participants’ accounts revealed some contradictions about a shared experience between individuals in a dyad. Contradictory stories came up on issues where one member said the opposite of the other member about an event. Below is an extract of field notes illustrating a contradictory story between an adolescent girl and her older carer:

“Today, I interviewed both B1 (older carer woman aged 64) and B2 (adolescent girl aged 14) separately for the first time. B2 was interviewed first. When asked about her experiences in the HIV prevention programme for adolescents and caregivers, B2 revealed that neither her grandmother (B1) nor anyone from the family attended the programme sessions with her. According to B2, she attended the programme alone without her caregiver. To our knowledge, the programme was designed for adolescents and their caregivers. Similarly, in the interview with B1, she also stated that she did not participate in the programme with B2. However, B1 differed from B2 in that B2 attended the programme sessions with her aunt [B1’s youngest daughter]”.

Probing contradictory stories was difficult as we were careful about asking direct questions, which could reveal the other dyad story, thereby breaching confidentiality. It was also not possible for the members to contest their contradicting stories as they were interviewed separately. This contradiction was not resolved; we accepted that each dyad member had a different version. Conforming to dyadic research, our focus was to look at perspectives on the same relational experience.

#### Single-sided account of a story

Methodological and ethical dilemmas were intertwined when a single-sided account of a story was experienced. This was when a dyad member presented information, which the other dyad member was discreet about. For instance, F1 (older carer aged 76) and E1 (older carer aged 56) were interviewed before their adolescent grandchildren. During the interviews, they both disclosed that they were living with HIV and on ART. The older carers shared that they had disclosed their HIV status to their adolescent grandchildren and that the adolescent grandchildren were supportive of the grandmothers to adhere to treatment. Interestingly, both the adolescent grandchildren F2 (adolescent girl aged 19) and E2 (adolescent girl aged 13) made no reference to their older carers’ HIV status during the interviews. The interviewer was caught in a dilemma about the extent of probing the second dyad member (adolescents) about the accounts of the first dyad member (older carers) while being mindful about the protection of confidentiality. Instead of trying to have adolescents’ talking about their older carer’s HIV status, we recognized that individuals had their story to tell in their own way. This also illustrated the nature of the care relationship.

### Challenges of interviewer-dyad interaction

#### Impossibility of matching age and gender characteristics of interviewer with dyads

All interviews were conducted in isiZulu by the first author, a middle-aged woman. The age and gender of interviewer did not match equally with all individual dyad participants. All the six older carers were women but older than the interviewer. Because of age differences, older carers viewed the interviewer as their daughter by addressing her as ‘*my child*’ or ‘*my daughter*’. On the contrary, the adolescents were younger than the interviewer hence they perceived the interviewer as ‘*a mother*’ or ‘*an aunt*’. These age and gender differences inevitably influenced the interaction between the participants and the interviewer. On the one hand, the age of interviewer facilitated and strengthened the relationship between the interviewer and older carers. On the other, during the first interviews, it was not easy for adolescents to explicitly express their experiences to an older interviewer whom they regarded as a mother. Adolescents tended to pause more often and for long periods and avoided eye contact with the interviewer. It is likely that the age of the interviewer may initially have been a barrier to the adolescents being open. Nonetheless, it was not feasible to cover the costs of a younger interviewer for subsequent interviews. Relying only on one interviewer renders age and gender matching impossible in dyadic research of this nature.

We took several measures to minimise the power differential between the interviewer and participants. Firstly, conducting repeat interviews with the same participant promoted deeper reflection and trust over the course of the data collection. Secondly, interviews took place in the participant’s home but after a period in which the interviewer would chat informally with the participant and observe their daily activities. This enabled greater access to the contextual details of their daily lives, including communication styles and behaviour patterns. The interviewer was thus better placed to integrate specific and detailed questions about the relational context of the participant’s lives. This investment in understanding the experiences of participants in their social context further consolidated trust and rapport. Lastly, to ameliorate the power differential between the interviewer and the adolescent participants, special attention was paid to avoiding controlling behaviours that might have led them to associate the interviewer with what might typically be expected of an interaction with a mother or aunt. Where possible the interviewer gave considerable autonomy to the participants, including initiating the scheduling of subsequent interviews so that they were conducted at a time that suited the adolescents and placing considerable emphasis on the control the participants could exert over whether and how they wanted to respond to any of the questions. All of this contributed to an overall investment in demonstrating how confidentiality was being maintained which further improved the trust between the adolescents and interviewer.

#### Unintended preferential treatment of participants by the researcher

While the interviewer spent a significant amount of time in the field to study the lives of the dyads within their naturalistic setting, unintended preferential bonding with individuals, particularly older carers, occurred. The interviewer established stronger rapport and more connections with the older carers, compared to the adolescents. Approaching older carers first and spending more time at the home waiting for the adolescents contributed to preferential bonding with the older carers. At every visit to the home, the interviewer had to announce her presence to the older carers first and to request permission to speak to the adolescents. Even when the adolescents knew the interviewer had come to see them, they waited to be called by their older carers that the interviewer had arrived for the interview with the adolescents. In addition, at the end of interviews with the adolescents, the interviewer had to announce her departure to the older carers. This is a social protocol in the area for home entry and home exit, and it is regarded as a symbol of respect to the elders or heads of families. As such, opportunities for interaction between the interviewer and older carers were greater than with the adolescents. During those interactions, the older carers always had important and interesting stories to tell the interviewer, also creating the need to follow-up their stories in subsequent visits. While the interviewer spent extended time with older carers and this generated detailed accounts about their worlds; however, it compromised adolescents’ voices as older carers’ views dominated the adolescents’ in the research process. Apart from complying with social protocols, our flexible interview structure allowed the interviewer to do the interview with any individual available between adolescents and the older carers. The interviewer also employed reflexivity as a tool to guide ethical practice throughout the study.

## Discussion

In this paper, we have shared our experience of conducting qualitative dyadic research with adolescent-older carer dyads in rural South Africa and presented ethical and methodological challenges, which have arisen, from our work. These include challenges on recruitment of dyads, consenting dyads, confidentiality, conducting separate dyadic interviews, and interviewer-dyad interaction. We also illustrated how we dealt with these challenges.

As guided by the relational agency, recruitment strategies are influenced by relationships between dyad members and the socio-ecological context of the research setting. Researchers must consider the cultural values of the participants they intend to recruit [34]. We recruited older carers first to align with socially accepted practices to engage with the caregivers prior to the young people. It was considered disrespectful to approach adolescents about participating in a research study without having first obtained permission from their caregivers. However, as other authors pointed out, this strategy may not address ethical concerns about the risk of coercion to participate among vulnerable populations [4, 5]. Different approaches could be employed to deal with ethical dilemmas arising from the recruitment of dyads. Young people can be approached first [5] or together with their caregivers [4]. In Uganda, young people nominated their caregivers and provided permission for researchers to approach caregivers [4]. By requesting children to identify their caregivers, it eliminates controlling behaviours from researchers and facilitates children empowerment in research by giving them a choice and a voice in the selection of dyad members.

Confidentiality is one of the cornerstones of research involving human participants. Protecting research participants’ right to confidentiality is a responsibility shared by researchers, institutional review boards, and participants themselves. Interviewing dyads within one intimate relationship posed three confidentiality challenges in this study: limited trust in the confidentiality process, unintended disclosure of information by the interviewer, and right to access confidential information of the other dyad member.

One strength of separate interviews is that it allows participants more freedom to express their individual perspective than in the absence of their partner [26]. Interviews with individual partners enabled them to reveal information to the interviewer while keeping it secret from their partner (e.g. pregnancy, sexual relationships). This enhanced our contextual understanding of the interpersonal relationships of dyads who kept secrets from each other [1]. Yet, some participants had limited trust in the confidentiality process, regardless of being interviewed separately. They anticipated that their information could be shared with their partners hence they insisted on assurance for confidentiality. Our finding reflects those of Allan who also found that individuals will reveal confidential information if they are certain it will never be disclosed to their partner [2]. In addition, the relational agency states that individuals’ experiences are shaped and influenced by their relationships with others [33]. An individual can have a sense of intentionally or unintentionally influencing another person in a constructive or deconstructive way [35]. In our study, the participants had a sense that if the information was disclosed to their partners, this could influence their relationships in a deconstructive way. While the adolescents may be narrating something to the interviewer, they sometimes appeared to be economising on what they disclosed to the interviewer perhaps because they are wondering about who will have access to their information. Certainly, in the first interviews, they may have wondered if they were speaking to the interviewer or speaking to their older carers by proxy as information might slip through the interviewer to the older carer.

Consistent with the literature, we found that, in separate interviews, participants expressed the desire to know what their partners said [25, 26]. A number of studies have begun to examine possible strategies for researchers to maintain confidentiality when conducting separate interviews with dyads [14, 25]. Our strategy reflects that of Zarhin [25] who also emphasized refraining from revealing information to protect participants’ confidentiality. In contrast to our strategy of reminding participants verbally about the confidentiality clause, Taylor and de Vocht [14] suggest incorporating this clause into the participant information sheets to make it clear to individuals that “no information shared by individuals would be disclosed to their partner.” However, attempting to be given the right to access information pertaining to their partners because of authority over the other is a relational issue. One’s actions and perceptions of self as an agent depends on the social and relationship context in which agency is enacted and experienced [35].

Critical to the success of dyad research is the confidence that the participants have in the confidentiality of the process, which relates not only to the data collection process but also to the presentation of findings. Securing this confidence is a process rather than a one-off activity. It can be secured as part of the initial informed consent process, but confidence may also be secured through the practice of confidentiality from recruitment to the dissemination of findings.

In this study, we found a single-sided account of stories between dyad members when interviewed separately. For example, older carers disclosed their HIV-positive status, which their adolescent grandchildren were discreet about. This finding was also reported by Norlyk and colleagues [27] when they conducted repeated interviews with patients and their partners who were living with Parkinson’s disease. They reported that ethical and methodological considerations were intertwined when one partner address an issue of interest and the other not [27]. Consistent with the literature, our study was unable to probe the specific reasons for the adolescents to be discreet about their older carers’ HIV status, as this may have breached the confidentiality of older carers’ information. The boundaries of individuals were respected because it was their decision about what and what not to share with the researcher [14]. Drawing from the relational agency, adolescents’ actions are influenced by the nature of the relationship with their carers, and the actions are a product of that relationship. As mentioned, the relational agency explains how adolescents’ experiences are shaped by their relationships and how their behaviours are influenced by the relational agency. Being silent about their older carers’ HIV-positive status might also illustrate how care might be done in these cases, through discretion. This shows the importance of listening to both sides by having separate dyadic interviews. Dyad members may experience the same event differently; thus, each individual’s story produces a dyad story, which in turn provides useful data to understand the dyad relationship. The fact of a single-sided account of a story reveals the nature of the relationship between individuals in the dyad. The implication of this tells us a little about how caring is bidirectional, but also can be hidden or not talked about for relational reasons. This methodological approach gives us valuable insights that may not have been revealed using a survey method or relying on only interviewing one individual within the dyad.

Therefore, the methodological and ethical challenges of the separate interviewing approach are associated with the relational agency. They are both a feature of the experience and a feature of the relational agency. Rather than to try to design a method, which resolves all these methodological and ethical dilemmas, it is important to recognise these challenges as features, or characteristics, of relationships and to illuminate that they exist. The challenges presented in this study displayed how things are experienced rather than them being the weaknesses of the method. They capture the essence of the experience of conducting a dyad study with adolescents and their older carers in our setting.

Although providing some very useful data on this under-researched issue, the major limitations of this study were the selection bias and the sample size. Prior to the study, we had planned to minimize the selection bias by creating a sampling frame of all adolescent recipients who were cared for by older carers using the implementing organization’s records. However, the implementing organisation did not have a database of adolescent recipients in the manner required to produce the sample frame of those in the care of older carers. The sample consisted exclusively of women older carers, which means that we should be cautious about interpreting methodological and ethical challenges in a dyadic approach involving different dyad groups. Although providing a unique perspective on ethical and methodological complexities in dyadic research with care dyads of adolescents and their older carers, research involving men older carers could develop and enhance our scientific knowledge.

## Conclusion

This paper demonstrates our experiences of methodological and ethical complexities associated with a qualitative dyadic approach in rural South Africa. It shows the difficulties concerning the recruitment of dyads, consenting dyads, maintaining confidentiality and conducting separate interviews with dyads; and illustrates how we dealt with the dilemmas. Despite methodological and ethical complexities, this approach allowed participants to share their intimate experiences, permitted adolescents’ accounts to be heard unmediated by older carers’ participation and has allowed a unique insight into how adolescents navigate their relationships with older carers. Lastly, we agree these ethical and methodological challenges should be recognised as features of relationships between dyads rather than weaknesses of the method.

## Data Availability

The datasets generated and analysed during the current study are available from the corresponding author on reasonable request.

## Abbreviations

AHRI: Africa Health Research Institute
LMCIs: Low-and-Middle-Income Countries
